# Theoretical study on β-cyclodextrin inclusion complexes with propiconazole and protonated propiconazole

**DOI:** 10.3762/bjoc.8.247

**Published:** 2012-12-17

**Authors:** Adrian Fifere, Narcisa Marangoci, Stelian Maier, Adina Coroaba, Dan Maftei, Mariana Pinteala

**Affiliations:** 1 Centre of Advance Research in Bionanoconjugates and Biopolymers, “Petru Poni” Institute of Macromolecular Chemistry, 700487 Iasi, Romania; 2 Faculty of Textiles & Leather Engineering and Industrial Management, “Gheorghe Asachi” Technical University of Iasi, 700050 Iasi, Romania; 3 Faculty of Chemistry, “Al. I. Cuza” University Iasi, Iasi 700506, Romania

**Keywords:** β-cyclodextrin, inclusion complexes, PM3, propiconazole

## Abstract

The synthesis of the β-cyclodextrin/propiconazole nitrate inclusion complex and the advantages of the encapsulation of this drug were recently reported, but the experimental data only partially revealed the structure of the supramolecular complex due to the limitations in understanding the intermolecular association mechanism. The present work describes the equilibrium molecular geometries of β-cyclodextrin/propiconazole and β-cyclodextrin/protonated propiconazole, established by the AM1 and PM3 semi-empirical methods. The affinity between different parts of the guest molecule and the cyclodextrin cavity was studied considering that propiconazole possesses three residues able to be included into the host cavity through primary or secondary hydroxyl rims. The results have revealed that the most stable complex is formed when the azole residue of the propiconazole enters the cavity of the cyclodextrin through the narrow hydroxyl’s rim.

## Introduction

The occurrence of fungal diseases has dramatically increased during the past 20 years. Extremely rare ten years ago, nowadays, antifungal drug resistance has become an important problem in treatment of fungal diseases for various categories of patients, especially those infected with HIV. Excessive and prolonged treatment with azole-containing medicines has led to fungal resistance to this class of compounds, especially in the case of HIV patients with repeated recurrent episodes [1–2]. Today, the number of reported cases of clinic resistance to antifungal drugs is growing and mycologists have warned about an increasingly large-scale expansion of this phenomenon [3]. Consequently, the development of new therapeutic conjugates able to combine new antifungal properties with water solubility of the drug has become a major direction of research in the field of antifungal therapy. In this respect, one of the expected methods consists of the complexation of antifungals with cyclodextrins and/or with soluble polymers.

Propiconazole (PP) is a triazole derivative effective as a fungicide, with a broad spectrum, designed and launched by Janssen Pharmaceutics (Belgium). It is widely used in agriculture as a systemic foliar fungicide and, lately, for its fungistatic action. Propiconazole nitrate was tested in order to reduce the toxicity of the unmodified PP, but little information is available on this topic. Recently synthesized positively charged protonated propiconazole (PPH^+^) showed an increased antifungal activity compared to unmodified PP. The inclusion compound based on β-cyclodextrin (β-CD) and PPH^+^ (further abbreviated as β-CD/PPH^+^) was preliminarily investigated in vitro, and its antifungal activity was reported [4].

Cyclodextrins (CDs) are macrocyclic oligosaccharides consisting of six to twelve glucopyranose units joined in a truncated cone-shaped structure [5]. They exhibit a hydrophobic cavity delimited by two rims, a wide and a narrow one, composed of secondary and primary hydroxy groups. By virtue of this structure, CDs are able to generate inclusion complexes with a wide variety of hydrophobic organic compounds in aqueous solution. The driving forces leading to complexation are numerous, varying from van der Waals to hydrophobic and to dipole–dipole interactions [6–7]. Since CDs and their complexes are widely used in pharmaceutical sciences and synthesis, there is currently a great interest in the theoretical study of their supramolecular associates.

Accurately representing the size of CD cavities and their chain flexibility represents a challenge for molecular simulation when quantum methods are employed. Since the ab initio approach is time consuming for this kind of molecule, quantum semi-empirical methods, such as CNDO, AM1 and PM3 were widely used in the theoretical investigation of CDs. The PM3 method has proved to be a powerful tool in the conformational study of supramolecular systems, such as CD inclusion compounds and provides better performance compared to the AM1 method for molecular geometry optimization, due to its improved description of hydrogen bonds and steric effects [8–10]. Advanced methods, such as Hartree–Fock (HF) and density functional theory (DFT), were also applied in cyclodextrin chemistry to explain experimental data [11–12]. Very often, ab initio methods are used in tandem with the semi-empirical PM3 method [13–17].

Because of the experimental limitations, the geometric details and the interactions that stabilize the molecular architecture of β-CD/PPH^+^ inclusion compounds are still poorly understood. For this reason the present study theoretically investigates the interaction between PP, PPH^+^ and β-CD molecules by means of AM1 and PM3 semi-empirical quantum-mechanical calculations, to examine in detail the insertion pathways and to determine the intimate configurations of the β-CD/propiconazole (β-CD/PP and β-CD/PPH^+^) inclusion complexes. Bearing in mind that the CD cavity cannot fully incorporate the guest molecule, the aim of this work is to identify the part of the molecular cavity that is more suitable for complexation with the PPH^+^. This information can be useful to predict which of the hydroxy groups of cyclodextrin can be chemically modified (by pegylation for example) in order to avoid the shielding of the cavity during the inclusion process, and, as a consequence, to improve the systemic bioavailability and pharmacokinetics of the inclusion complexes.

## Results and Discussions

**Figure 1:**
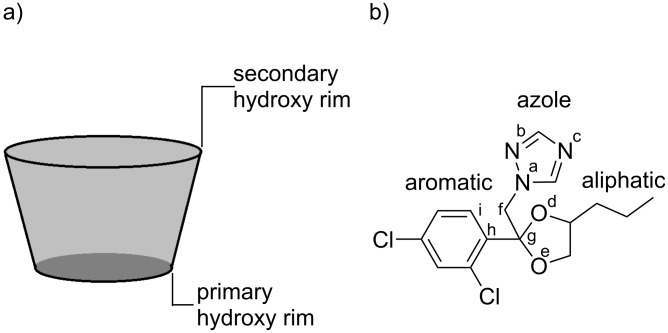
Schematic representation of the β-cyclodextrin (a) and propiconazole (b) molecules.

The most stable conformations of the β-CD/PP and β-CD/PPH^+^ inclusion compounds were selected by considering the binding energy as being the difference between the heat of formation of the complex and the heat of formation of the involved free molecules:

[1]
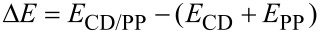


where *E*_CD/PP_, *E*_PP_ and *E*_CD_ represent the heat of formation of the complex, of the free β-CD, and of the free guest molecule, respectively. The higher the negative value of the stabilization energy, the more thermodynamically favorable is the pathway of inclusion-complex formation. The particular shape of the PP molecule allows its inclusion into the β-CD cavity following three different ways. Additionally, each residue of PP can be well accommodated either by the secondary or the primary face of the β-CD cavity (Figure 1a). Therefore, six configurations must be considered in pursuing the most stable molecular structure of the inclusion complex. For simplicity, let us note each PP residue as it is shown in Figure 1b. The orientation of PP toward β-CD will be named according to the PP residue that is first included through the wider (A) or narrower (B) cavity rim (e.g., aliphatic A, aliphatic B, if the aliphatic residue is included through the wider or narrower hydroxy rim of the cyclodextrin). A suitable methodology for finding the equilibrium molecular geometry of cyclodextrin inclusion complexes is to place the guest on the cavity axis and to move it through the cavity in steps, simultaneously optimizing the conformations. Since there are six possible configurations, it is crucial to find which propiconazole residue has maximum compatibility with the cyclodextrin cavity. To save computational resources, this can be achieved by the methodology described in the computational method section of the paper. The first step in attaining this goal involves the setting of some conformational constraints, followed by the energetic minimization of the resulted conformation. Hence, the optimized molecular geometries will contain the guest molecule inside or outside of the β-CD, depending on the molecular hindrance and on the affinity between the guest residues and CD cavities. Finally, the structures are to be subjected to PM3 calculations, without any constraints, to obtain the heats of formation and to compare the stability of the conformers.

**Figure 2:**
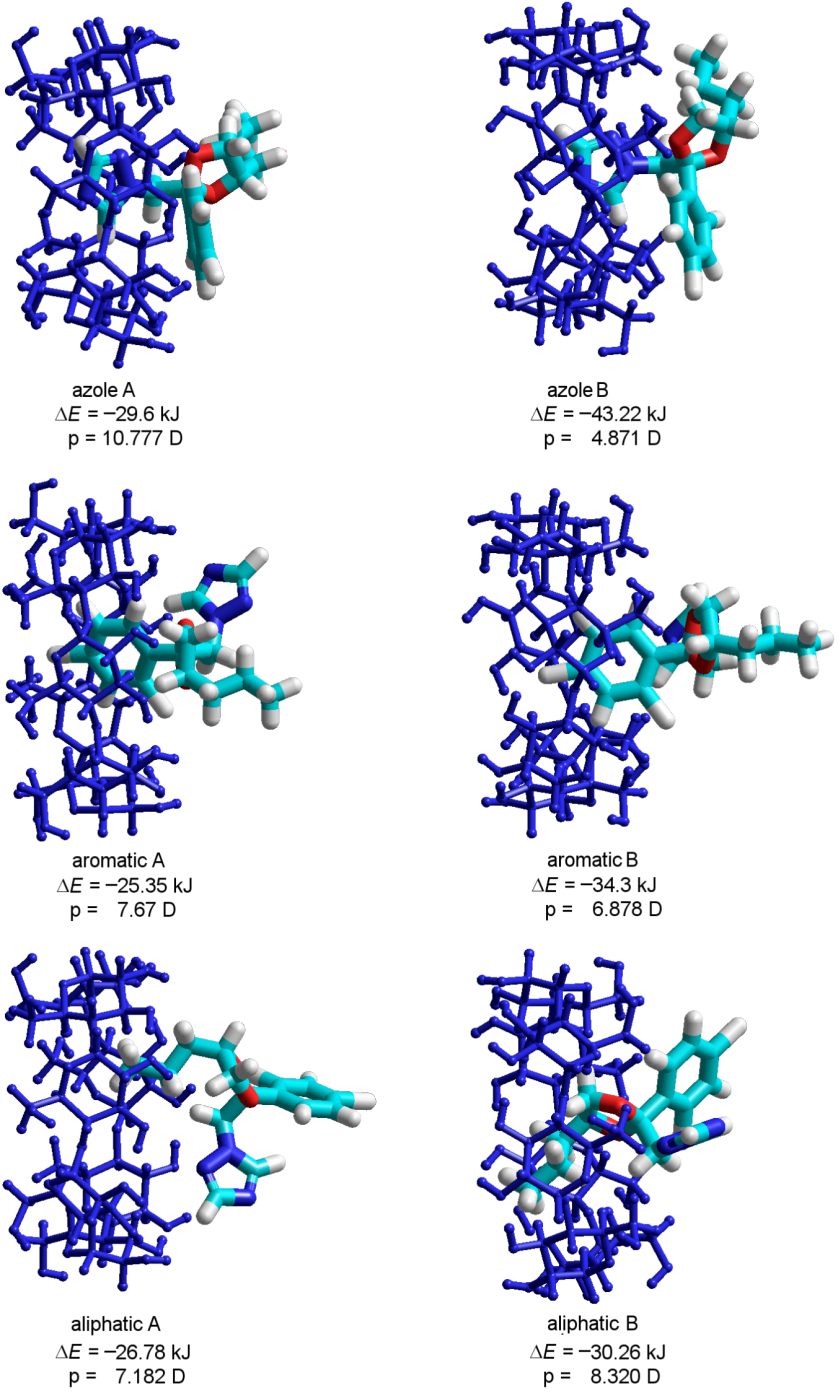
PM3 optimized molecular geometries of the β-CD/PP inclusion compounds involved in the assessment of PP inclusion into the β-CD cavity by each of its residues.

Looking at Figure 2 it is obvious that all structures are stable since, in all cases, negative binding energies were obtained. It can also be observed that the PP deeply entered into the cavity during the calculation, demonstrating a high probability of complex formation. The binding energy is not very high, which could be explained by the absence of hydrogen bonds that strongly stabilize the molecular association. Analyzing the numerical values summarized in Figure 2, one can see that the stability of the complexes is inversely related to their global electric dipoles. There is an obvious correlation between the global electric dipole moments (p) of the complexes and their binding energies, for each paired situation including the PP residues. Such a fact suggests that a dipole–dipole coupling mechanism could be involved in the complex formation. The inclusion of the triazole ring is energetically favored, since the resulting binding energy is the lowest. The obtained theoretical results confirm the experimental data published on complexation of β-CD and PP, which highlights the inclusion of the triazole ring in the cyclodextrin cavity [4]. Hence, the developed interaction model is accurate.

### Inclusion compounds with non-protonated propiconazole (β-CD/PP)

**Figure 3:**
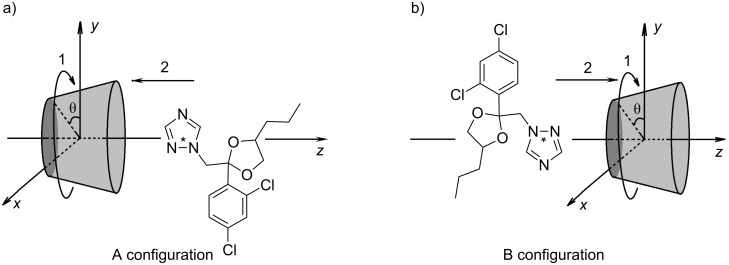
Molecular coordinates used to describe the relative position between the β-CD and guest molecules.

In order to determine the exact structure of the obtained complex, further calculations were carried out. Taking into account previous theoretical and experimental results, we assumed that the β-CD/PP complex is formed by triazole ring inclusion into the cyclodextrin cavity. The further study was performed considering two orientations of the triazole ring in relation to the CD cavity: one with the triazole ring pointing toward the negative sense of the *z* axis, denoted by A (Figure 3a), and the other with the triazole ring pointing toward the positive sense of the cavity axis, denoted by B (Figure 3b). The intermolecular distance was measured between the cavity center and the “dummy” atom of the triazole ring (marked with an asterisk). Initially, the triazole ring was placed in the center of the cavity and a complete rotation was performed to establish the preferred angular orientation of PP during the inclusion process. Keeping constant the resulting angular orientation, PP was then moved along the *z* axis simultaneously with geometry optimization, in the absence of any symmetry constraints (Figure 3a and Figure 3b) as discussed above.

**Figure 4:**
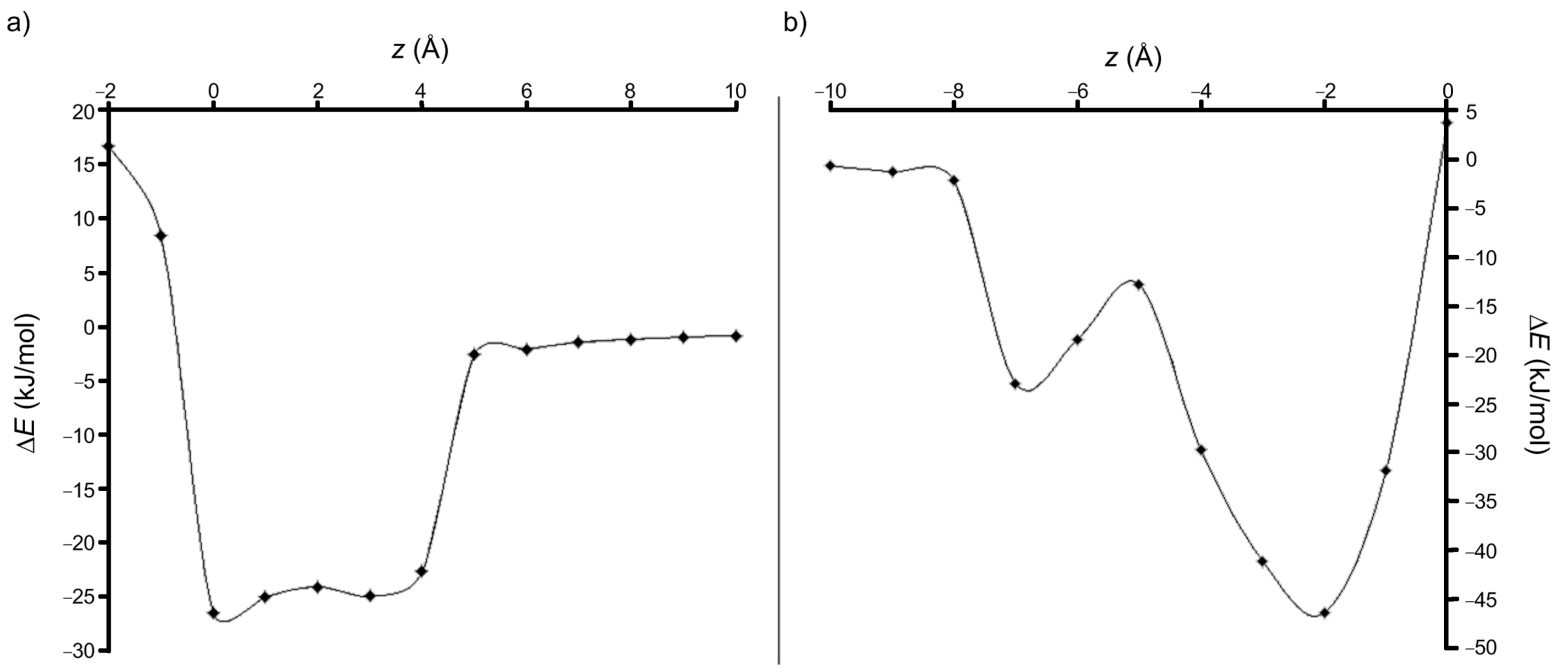
Evolution of the stabilization energy during the movement along the *z* axis in the case of (a) A and (b) B orientations of PP relative to the cavity of β-CD (PM3 calculation).

Scanning the binding energy during the movement along the *z* axis, by using the PM3 method, always provided negative values for both A and B configurations (Figure 4a and Figure 4b).

**Figure 5:**
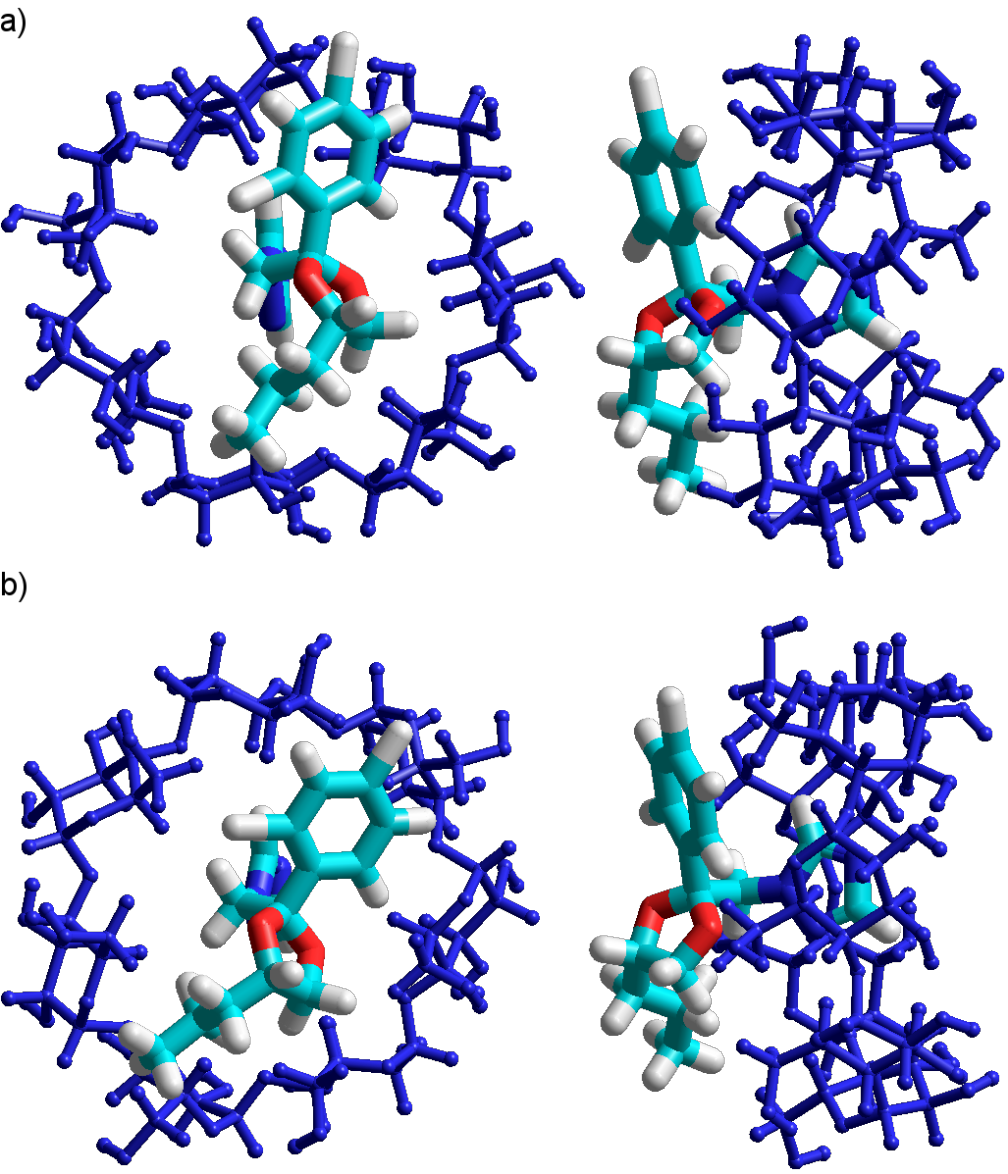
PM3 optimized molecular geometry of the β-CD/PP inclusion compounds in (a) A configuration and in (b) B configuration.

The most stable equilibrium molecular geometry is obtained when PP is deeply included in the β-CD cavity, suggesting an enthalpically driven process (Figure 5a and Figure 5b). The obtained results confirm that PP penetration through the rim of the primary hydroxy is thermodynamically favored, and that the complex is stabilized in the B orientation, which has a stabilization energy about ~20 kJ smaller as compared to the A configuration (Table 1). These facts are in good agreement with those exposed in Figure 2, confirming a higher probability of azole ring inclusion into the β-CD cavity through the narrow rim, according to the B configuration. The results are also consistent with the DFT single-point computations applied on the PM3 equilibrium geometries.

**Table 1:** Molecular parameters of the most stable β-CD/PP inclusion compounds in both A and B configurations, as given by AM1 and PM3 calculations and by B3LYP/6-31G(d)+ single-point calculations applied on the PM3 optimized geometries.

Parameter	Method of calculation^a^	β-CD (kJ/mol)	PP (kJ/mol)	β-CD/PP A configuration	β-CD/PP B configuration

*E* (kJ/mol)	AM1	−6895.59	21.08	−6879.92	−6889.54
	PM3	−6091.27	−91.71	−6209.44	−6229.34
	DFT	−11224754.47	−4770267.2	–	–
Δ*E* (kJ/mol)	AM1	–	–	−5.40	−15.02
	PM3	–	–	−26.45	−46.35
	DFT	–	–	−29.39	−49.64
p (D)	AM1	5.028	3.613	7.36	1.037
	PM3	6.915	3.945	9.633	4.644
	DFT	8.181	4.034	10.987	5.521

^a^In the case of DFT calculations, total energies were taken into account.

The results are similar to those reported by Fatiha [18], where the imidazole ring of sulconazole enters through the narrow rim into the β-cyclodextrin cavity. The β-CD/sulconazole is stabilized by van der Waals interactions and hydrogen bonds. In fact, the author explained the difference between the A and B orientations in the case of sulconazole by the occurrence of hydrogen bonds. In the present case, no hydrogen bonds were evidenced and, since there are no steric constraints to explain the difference between the A and B orientations in terms of stability, other forces must be considered. It is already known that cyclodextrin molecules have a rather high electric dipole moment; the correlation between this parameter and the complex stability by means of quantum-mechanical calculations has been previously reported [19–20]. The PP molecule has a permanent electric dipole, and the dipole–dipole interaction can make the difference between the A and B configurations. The decrease of the global dipole moment together with the increase of the binding energy (see Table 1), points to the dipole–dipole interactions as a major contributor to the stabilization of the B structure.

**Figure 6:**
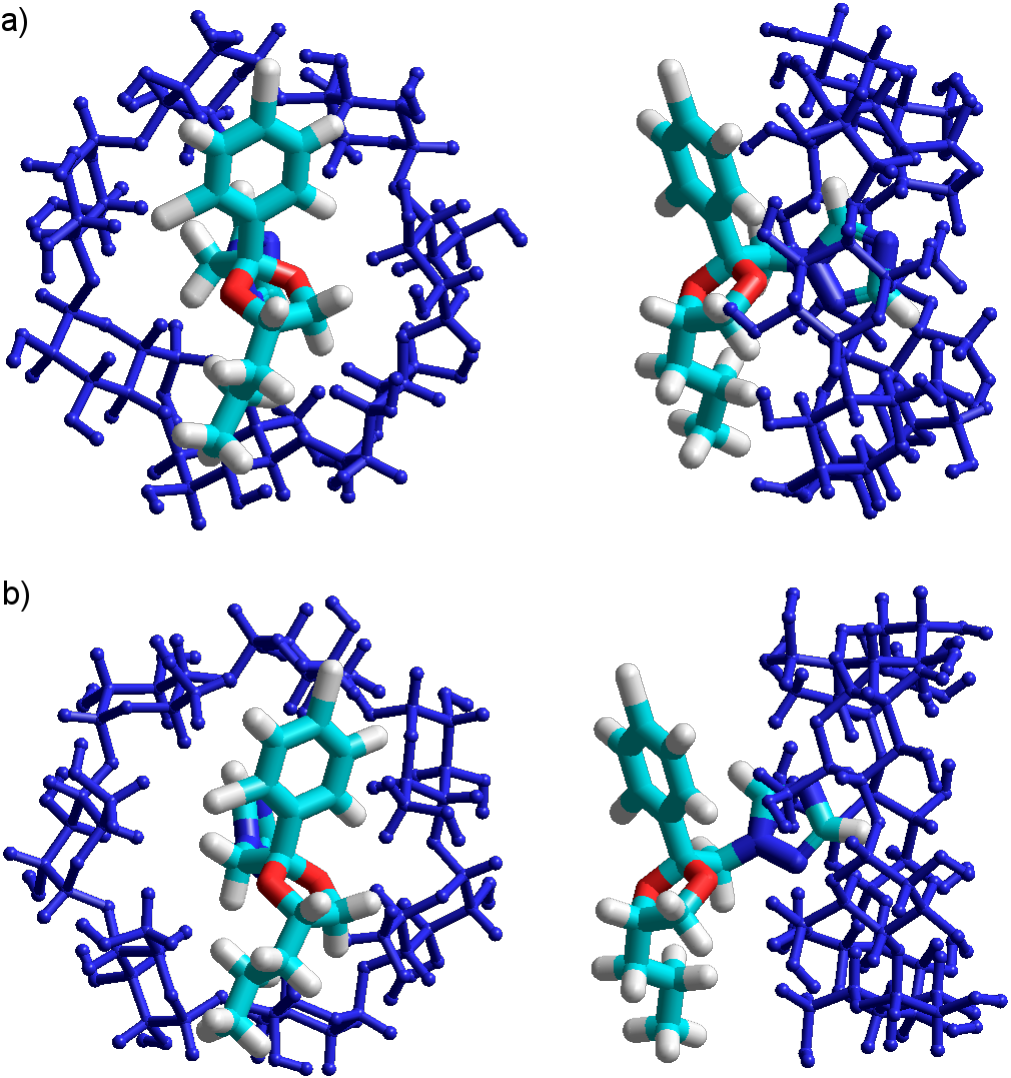
AM1 optimized molecular geometry of the β-CD/PP inclusion compounds, for both (a) A and (b) B configurations.

As depicted in Figure 6a and Figure 6b, the molecular geometry of the AM1 optimized inclusion compounds shows that the PP molecule is deeply inserted in the cavity of β-CD for both A and B configurations.

In terms of binding energy, the difference between the A and B orientations was found to be 9.62 kJ. Similarly to the preceding PM3 calculation, the AM1 optimized B configuration of the β-CD/PP complex has a higher stability and no hydrogen bonds where identified. After AM1 optimization too, the same correlation between the stabilization energies and dipole moments of the inclusion compounds was revealed (Table 1).

### Inclusion compounds with protonated propiconazole (β-CD/PPH^+^)

In order to study the complex formation between β-CD and the positively charged guest molecule, a model of PPH^+^ was built. The proton affinity (PA) was calculated for each of the five protonation sites of the azole ring (according to the notation scheme shown in Figure 1b). According to reference [21–22], PA was defined as the energy variation in the proton-addition equilibrium:

[2]
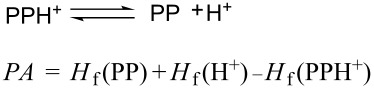


In this model the heat of formation of the proton was taken to be zero because of the lack of electrons, and the reaction system was considered in vacuo, at 0 K. The method does not provide the exact value of the PA parameter, but the calculation is useful to compare the stability of different protonation forms of the PP molecule. Table 2 shows that the PA values are considerably higher when PP is protonated at the iminic nitrogen atom (in the c position), this position being therefore the best protonation site.

**Table 2:** The calculated values of the proton affinity of the denoted atoms in the PP molecule (see Figure 1b).

PPH^+^	PA (kJ/mol)

PPH^+^ a	758.05
PPH^+^ b	848.22
PPH^+^ c	907.99
PPH^+^ d	734.36
PPH^+^ e	760.32

To find the energy-minimized molecular geometry of the β-CD/PPH^+^ complex, only the inclusion of the protonated azole ring into the β-CD cavity was considered. The procedure was similar to that used in the case of the β-CD/PP inclusion complex, the azole ring being progressively introduced into the β-CD cavity. Also, similar to the β-CD/PP complex, two orientations of the guest molecule relative to the β-CD cavity were taken into account and the same notations are kept.

**Figure 7:**
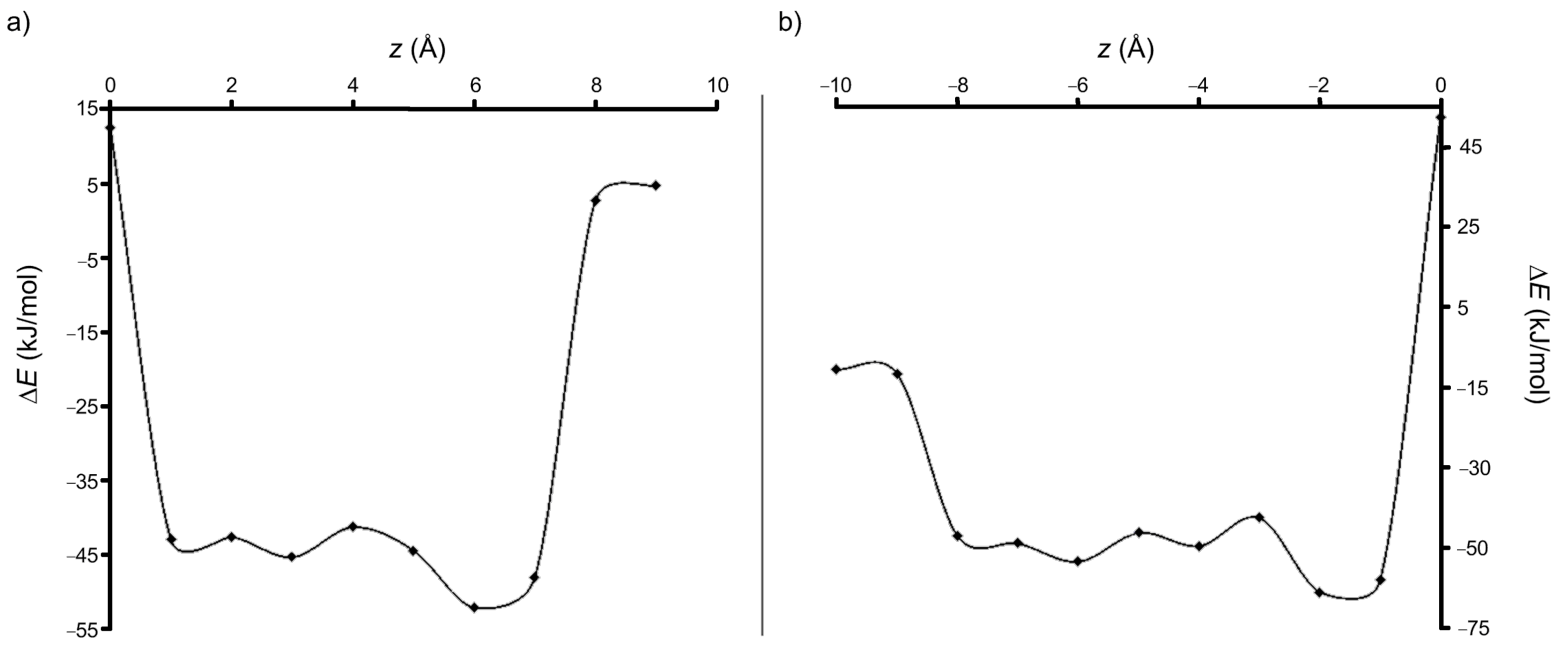
Variation of the stabilization energy during the movement along the *z* axis, in the case of (a) A and (b) B orientations of PPH^+^ relative to the β-CD cavity (PM3 calculations).

Scanning the binding energy by using the PM3 method revealed that, for all step intervals along the *z* axis, the energy of the complex is substantially lower compared with the sum of the energies of the isolated host and guest molecules (Figure 7a and Figure 7b). According to the values of the energy of the two configurations, it results that the B orientation of the PPH^+^ molecule relative to the β-CD cavity provides the most stable inclusion complex, the difference being about 13.8 kJ/mol when compared to the A configuration (Table 3). Results are also consistent with the DFT single point computations applied on the PM3 equilibrium geometries.

**Table 3:** Molecular parameters of the most stable β-CD/PPH^+^ inclusion compounds in both A and B configuration, as given by AM1 and PM3 calculations and by B3LYP/6-31G(d)+ single-point calculations applied on the PM3 optimized geometries.

Parameter	Method of calculation^a^	β-CD	PPH^+^	β-CD/PPH^+^ A orientation	β-CD/PPH^+^ B orientation

*E* (kJ/mol)	AM1	−6895.59	150.66	−6308.95	−6352.23
	PM3	−6091.27	537.16	−5606.27	−5620.07
	DFT	−11224754.47	−4771227.68	–	–
Δ*E* (kJ/mol)	AM1	–	–	−44	−87.28
	PM3	–	–	−52.16	−65.96
	DFT	–	–	−28.63	−38.61
p (D)	AM1	5.028	13.077	17.476	1.09
	PM3	6.915	12.086	19.531	7.123
	DFT	8.181	10.898	10.987	7.145

^a^In the case of DFT calculations, total energies were taken into account.

**Figure 8:**
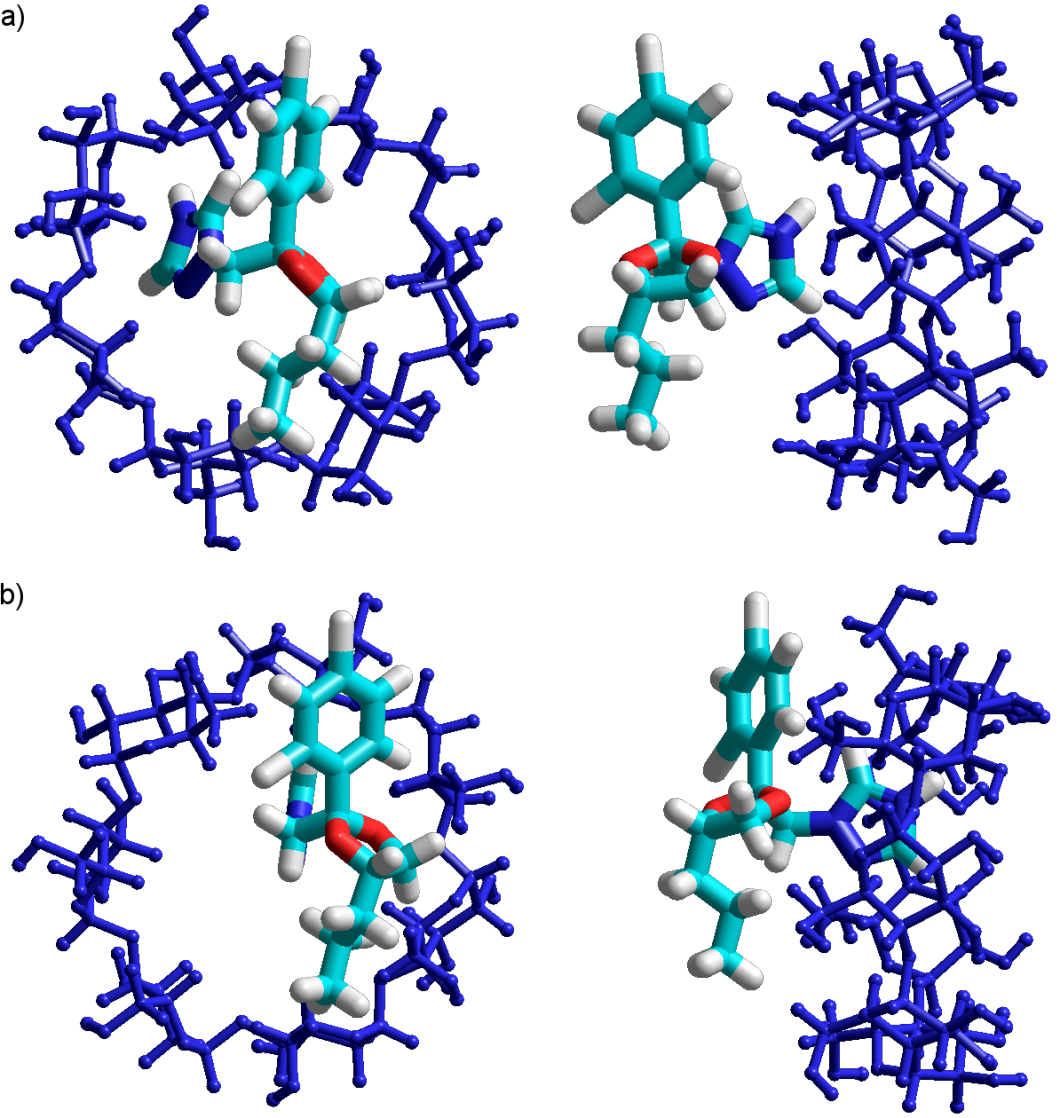
PM3 optimized molecular geometry of β-CD/PPH^+^ inclusion compounds in the (a) A and (b) B configurations.

As depicted in Figure 8a and Figure 8b, the equilibrium molecular geometries of the A and B PM3 optimized inclusion compounds are quite different. In the A orientation, the stable complex is formed with the protonated azole ring located outside of the β-CD cavity (Figure 8a), while in the B orientation, the azole ring is completely included in the cavity (Figure 8b).

**Figure 9:**
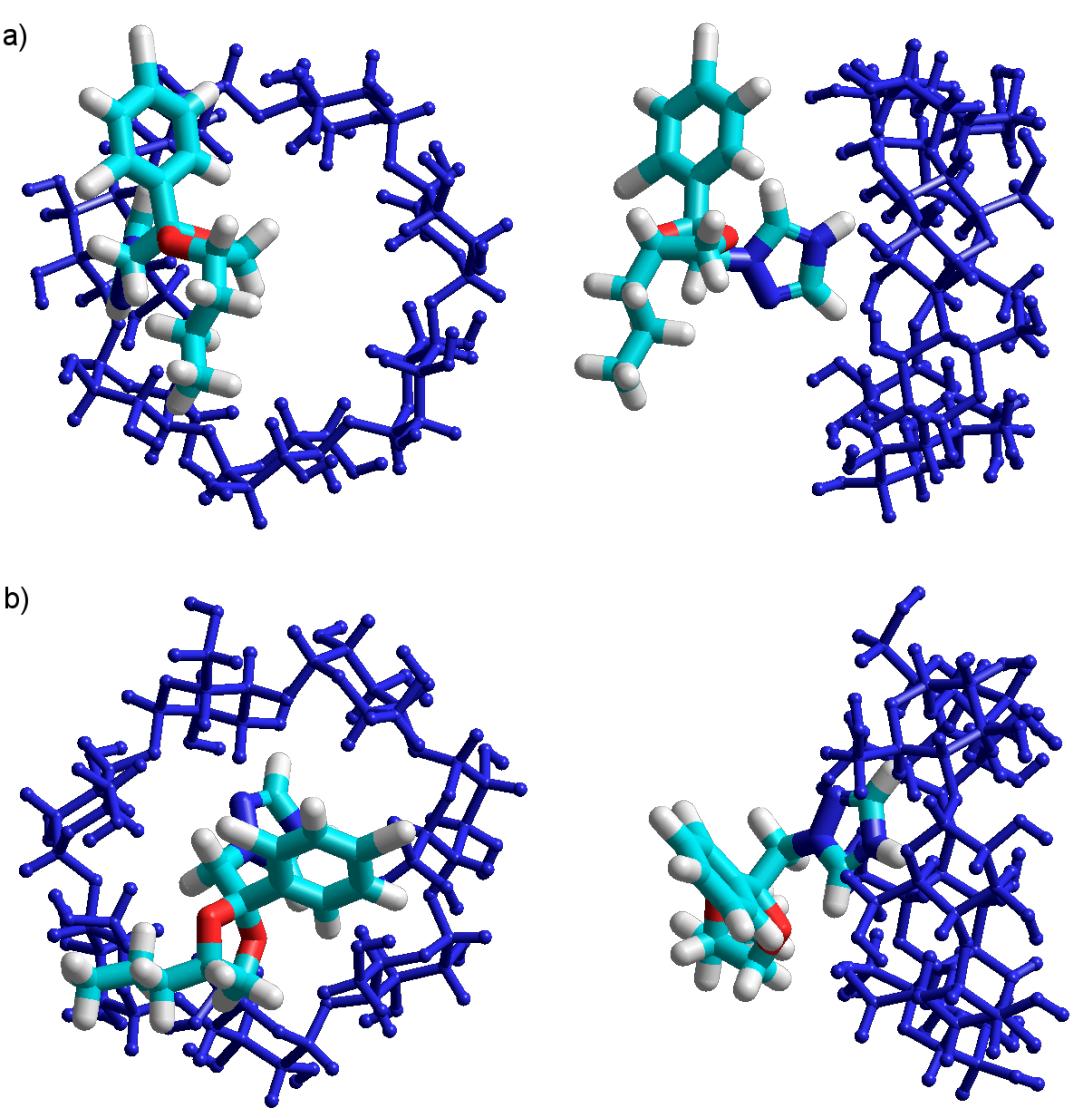
AM1 optimized molecular geometry of the inclusion compounds β-CD/PPH^+^ in the (a) A and (b) B configurations.

The AM1 method shows the same trend as the PM3 regarding the inclusion pathway of PPH^+^ in the β-CD cavity, where the complex has the highest stability in the B configuration. The binding energy of the B configuration is 43.28 kJ/mol lower than that in the A configuration (Table 3). The significant difference between stabilization energies leads to a rather big difference between molecular structures. Thus, while in the more stable B configuration the PPH^+^ is included with the azole ring in the β-CD cavity, at the level of the wider rim (Figure 9a), in the A configuration, the PPH^+^ molecule is completely outside of the cavity (Figure 9b). Both AM1 and PM3 calculations indicate a strong correlation between the stabilization energy and the electric dipole moments (Table 3).

Modeling the inclusion pathway of PP and PPH^+^ by means of semi-empirical PM3 and AM1, a strong correlation between the values of binding energy and electric dipole moments is revealed, which indicates a major contribution of dipole–dipole coupling to the molecular stability of the inclusion complexes.

The energy minimization of the β-CD/PP and β-CD/PPH^+^ inclusion compounds with the PM3 and AM1 methods leads to equilibrium geometries with the guest molecules partially inserted in the β-CD cavity for both A and B orientations of the guests. When the protonated form of PP is considered, the process minimization generates very different molecular architectures for the A and B starting configurations. While the complex in the A configuration contains the PPH^+^ molecule completely outside of the β-CD cavity, in the case of the B configuration, PPH^+^ is deeply inserted in the cavity with the protonated azole ring entering through the narrow rim. By protonating the PP, a regional selectivity of the inclusion process relative to the β-CD cavity was noticed, with the complexation path through the narrow rim being the favored one.

As Table 4 shows, PP and PPH^+^ experience some conformational transformations during the complexation process. The variation of molecular parameters is not significant, but it seems that the PP molecule changes its shape in order to augment the complex stability. The magnitude of the variation is higher for the β-CD/PP complex than for the β-CD/PPH^+^ one, and is significant at the level of dihedral angles of the molecules. In the case of the β-CD/PP complex, the magnitude of the variation of the molecular parameters is higher for the A orientation, while in the case of β-CD/PPH^+^ the trend is reversed. Since for both PP and PPH^+^, the most stable complex occurs in the B orientation, and because the magnitude of the conformational change is not correlated with any of the two configurations, we suppose that regional selectivity of cyclodextrin complexation is not governed by steric driving forces or by conformational effort of the PP during the inclusion process.

**Table 4:** Relevant geometric parameters of the PP and PPH^+^ molecules in their inclusion compounds with β-CD, as given by PM3 calculations.

Molecular parameter	PP	β-CD/PP A	β-CD/PP B	PPH^+^	β-CD/PPH^+^ A	β-CD/PPH^+^ B

Bond N_a_–C_f_	1.47	1.47	1.47	1.47	1.47	1.47
C_f_–C_g_	1.56	1.56	1.56	1.57	1.57	1.57
C_g_–C_h_	1.52	1.52	1.52	1.52	1.52	1.523

Angle N_a_–C_f_–C_g_	114.18	114.23	114.27	113.90	113.85	114.31
C_f_–C_g_–C_h_	110.82	111.25	110.68	111.1	111.01	111.77
C_f_–C_g_–O_d_	110.81	110.29	110.89	108.17	108.68	107.84
C_g_–C_h_–C_i_	120.46	120.34	120.80	120.54	120.50	120.27

Dihedral angle N_a_–C_f_–C_g_–C_h_	74.68	86.61	71.29	73.60	72.08	78.69
N_a_–C_f_–C_g_–O_d_	−48.92	−37.25	−52.19	−50.31	−51.77	−45.52
C_f_–C_g_–C_h_–C_i_	−110.26	−112.83	−110.56	116.23	−114.14	−119.59

### The effect of solvent on the orientation of a guest molecule toward the β-CD cavity

Since all the previously discussed molecular geometries were optimized in vacuo, it is important to know if the orientation of the guest molecules toward the β-CD cavity remains the same in the presence of water molecules. In this respect, the geometries of the complexes were optimized in aqua, by combining techniques of molecular mechanics and quantum mechanics [23].

**Figure 10:**
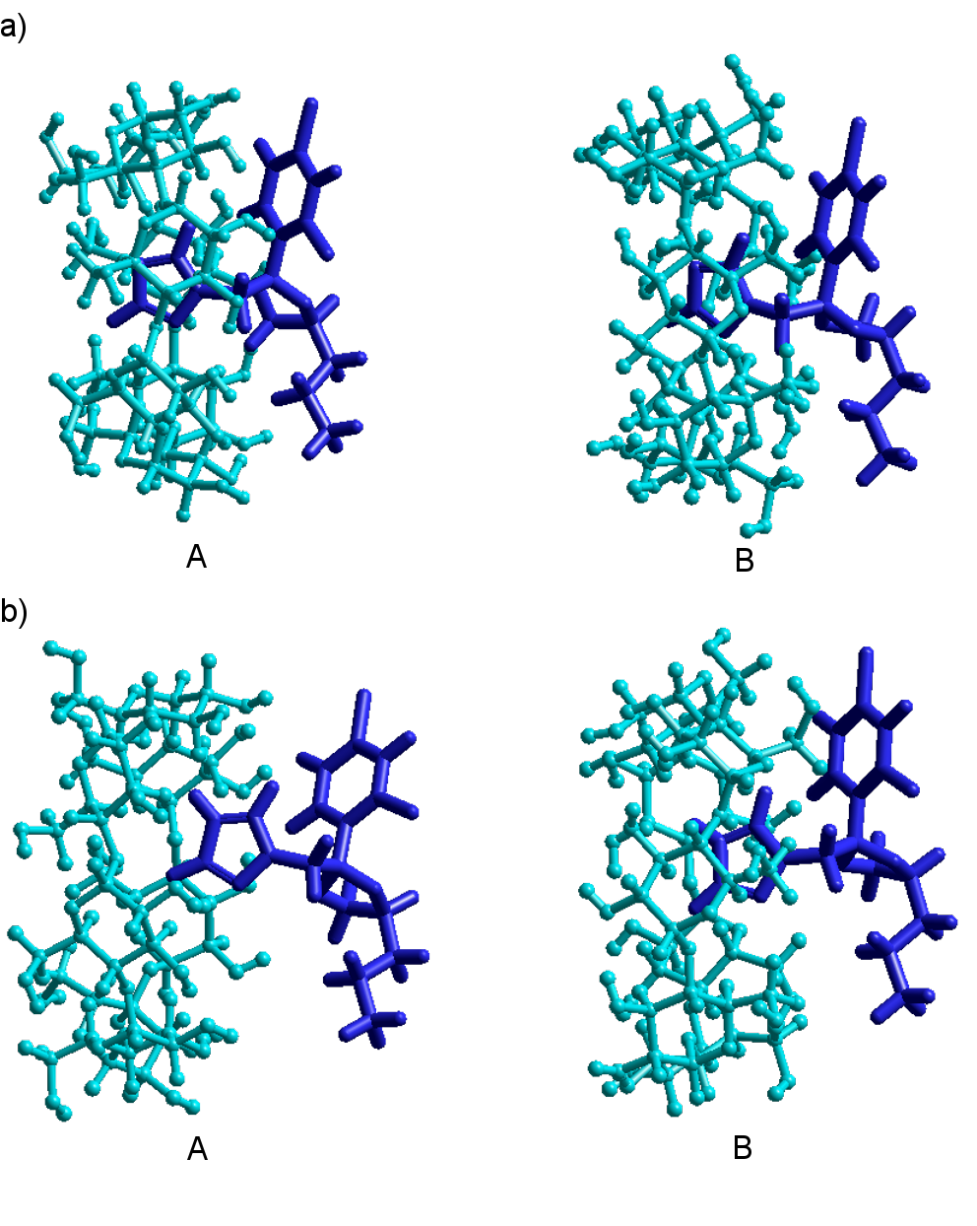
MM+ optimized molecular geometry of the (a) β-CD/PP and (b) β-CD/PPH^+^ inclusion complexes, in both A and B configurations. For clarity, water molecules have been removed.

The results given in Table 5 shows that, as solvent, water does not affect the most stable orientation of PP and PPH^+^ toward the β-CD cavity, and that the binding energy permanently remains negative. Likewise with the case of in vacuo calculations, the B orientations of the guests are favored for both β-CD/PP and β-CD/PPH^+^ inclusion complexes. It is important to note that both in vacuo and in aqua, the guest molecules are deeply inserted in the β-CD cavity, according to the B configurations (Figure 10), and no hydrogen bonds can be evidenced. The decrease of the global dipole moment by the increase of binding energy, suggests again that dipole–dipole interactions are the major contributor to the stabilization of the B structure.

**Table 5:** Molecular parameters of the most stable β-CD/PP and β-CD/PPH^+^ inclusion complexes in A and B configurations, as given by the in aqua PM3 calculations.

Parameter	β-CD/PP		β-CD/PPH^+^	
	A orientation	B orientation	A orientation	B orientation

Δ*E* (kJ/mol)	−11.4	−17.1	−12.15	−34.44
p (D)	10.587	4.278	20.429	4.559

## Conclusion

The molecular geometry of the inclusion complexes of β-CD with PP and PPH^+^ as guests was studied by using the MM+, AM1 and PM3 methods. The results have revealed that the PP and PPH^+^ azole rings were included into the cavity of the β-CD through the narrower hydroxy rim. A strong correlation between the binding energy and the global dipole moments was proved, pointing to the fact that dipole–dipole coupling acts as a major force in stabilizing the complexes.

The presence of water molecules as a solvent does not affect the orientation of PP and PPH^+^ toward the β-CD cavity: both guests penetrating through the narrow rim is always favored. The strong correlation between the binding energy and the global dipole moment is obviously maintained, no matter if the inclusion process is simulated in vacuo or in the presence of water molecules.

## Experimental

### Computational method

The starting molecular conformations of β-CD, PP and PPH^+^ were built by using the graphical tool of the HyperChem 7.52 software application [24]. β-CD was built up starting from α-D-glucopyranose residues (found in HyperChem data base) by interconnection with α-(1,4)-glycosidic oxygen bridges. The resulting molecular geometries where fully optimized by AM1 and PM3 quantum-mechanics semi-empirical methods, under HyperChem software application. DFT single-point calculations were performed using GAUSSIAN 09 software package [25], at the level of B3LYP/6-31G(d)+. The equilibrium molecular geometries were validated by comparing the obtained molecular parameters with those reported in the literature [10].

It is known that the calculation of the entire potential surface of CD inclusion complexes requires large computational resources and is time consuming. Because the three entering pathways of the PP molecule into the molecular cavity of the β-CD take place according to complexation mechanisms, several methods were developed to prove the compatibility of the molecular residue of the guest with the host cavity [26–29].

A molecular coordinate system was defined having the glycosidic oxygen atoms in the *x*O*y* plane, and with the *z* axis orthogonal to *xy* plane, so that *z* axis becomes the cavity axis. The primary hydroxy groups of cyclodextrin are therefore oriented toward the negative sense of *z*, while the secondary ones are directed toward the positive sense of the axis. In our approximation, the inclusion processes take place with the guest molecule moving along the cavity axis. To determine which PP residue has the highest affinity for the β-CD cavity, the PP molecule was placed on the cavity axis with one of the residues (either aromatic, aliphatic or azole ring) in front of the β-CD cavity. As a first stage, a MM+ calculation was applied to the guest PP molecule, while keeping the β-CD conformation frozen. The result was a deep insertion of the PP into the cavity, along with the advance of the calculation process. In the next stage, the whole system was fully optimized by using the PM3 quantum method, without any conformational constraints.

The molecular equilibrium geometries of the β-CD inclusion compounds with PP and PPH^+^ were calculated by placing the drug molecule on the cavity axis. To find the favorable angular orientation, the guest molecule was placed in the center of the cavity and a complete rotation was performed, optimizing the molecular geometry by PM3 methods at equal intervals of 20°, in the absence of any conformational constraints. While keeping the favorable angular orientation constant, the guest was moved along the cavity axis through the β-CD cavity, and complete geometry-optimization calculations were performed for equal intervals of 1 Å, by the PM3 method.

To compare the results delivered by the two semi-empirical methods, the molecules with initial coordinates corresponding to their PM3 minimum energy were subjected to AM1 calculations and also to B3LYP/6-31G(d)+ single-point investigations.

The most stable inclusion complexes found by PM3 computations were placed in a cubic box with 36 × 36 × 36 Å, containing 1541 water molecules. The systems were firstly equilibrated by optimizing water molecules with the MM+ method, keeping the inclusion complexes frozen. Then a complete optimization of the whole system was performed for each system, without any constraints [23]. The resulting molecular geometries were finally subjected to singlepoint calculation by the PM3 method, in order to extract the heat of formation, after the removal of water molecules. A full optimization of the equilibrium geometries after removing water molecules would have led to a complete loss of information concerning the solvent effect.
